# Parental Obesity, Lifestyle Factors and Obesity in Preschool Children: Results of the Toyama Birth Cohort Study

**DOI:** 10.2188/jea.12.33

**Published:** 2007-11-30

**Authors:** Michikazu Sekine, Takashi Yamagami, Shimako Hamanishi, Kyoko Handa, Tomohiro Saito, Seiichiro Nanri, Katsuhiko Kawaminami, Noritaka Tokui, Katsumi Yoshida, Sadanobu Kagamimori

**Affiliations:** 1 Department of Welfare Promotion and Epidemiology, Toyama Medical and Pharmaceutical University.; 2 Takaoka Public Health Center.; 3 Division of Environmental Epidemiology, National Children’s Hospital.; 4 Health Center, Keio University.; 5 Department of Epidemiology, National Institute of Public Health.; 6 Department of Clinical Epidemiology, University of Occupational and Environmental Health.; 7 Department of Preventive Medicine, St. Marianna University.

**Keywords:** child, parent, sleep, obesity, cross-sectional study

## Abstract

The aim of this study was to clarify the impact of parental obesity and lifestyle factors on obesity in preschool children. The subjects consisted of 8941 children aged 3 years, born in 1989. Anthropometric measurements and questionnaire surveys were conducted between 1992 and 1994. Subjects of body mass index (BMI; (weight (kg)) / (height (m))^2^) more than the age- and sex-specific centiles linked to adult overweight were defined as obese subjects. Parental obesity was defined as BMI of 25 kg/m^2^ or more. Logistic regression analysis was performed to clarify the strengths of parental and lifestyle factors on childhood obesity, adjusted for possible confounding factors. Odds ratios (ORs) of paternal and maternal obesity for childhood obesity were 1.70(1.43-2.02) and 2.56(2.07-3.17), respectively. There was a dose-response relationship between short sleeping hours and obesity. Compared to subjects taking 11 hours sleep or more, the adjusted OR was 1.20(0.97-1.49) for those taking 10 to 11 hours sleep, 1.34(1.05-1.72) for those taking 9 to 10 hours sleep, and 1.57(0.90-2.75) for those taking less than 9 hours sleep. Eating and exercising habits were not significantly associated with obesity. These results indicate that parental obesity and short sleeping hours are possible risk factors for obesity in preschool children.

## INTRODUCTION

The increase in childhood obesity is a major public health concern in developed countries^1^^,^^2^^)^. In Japan, the prevalence of childhood obesity increased to 8-12% in the 1990’s, which is 3 times higher than that in the 1970’s^2^^)^. Because of the rapid increase in childhood obesity over a relatively short period of time, many researchers have focused on the relationship between lifestyle factors and the development of obesity^3^^-^^5^^)^.

To clarify the impact of lifestyle factors on childhood obesity, we are conducting a birth cohort study of children born in 1989 in Toyama prefecture^6^^-^^8^^)^. To date, we have published three studies regarding the relationship between lifestyles and obesity in preschool children. Two studies^7^^,^^8^^)^ were case-control studies using cross-sectional collected data, in which the cases were subjects with a body mass index (BMI; weight in kilogram divided by squared height in meters) of 18 kg/m^2^ or more and controls were subjects with a BMI of less than 18 kg/m^2^, matched for sex and month of birth. The other study^6^^)^ was a cross-sectional study in which a BMI of 18 kg/m^2^ or more was used as the cutoff point for childhood obesity. Because there was no established cutoff point for obesity in preschool children, these previous papers employed a BMI obesity criterion of 18 kg/m^2^ or more, which was commonly used at that time^9^^)^. Although our initial survey was based on the health checkup for 3-year-old children, according to the Law for the Health of Mothers and Children, the subjects’ age at entry ranged from 2.5 to 4.3 years. The height and weight of children changes dramatically even in one year. For instance, the average BMI decreases with age in preschool years, which results from a decrease in baby fat^10^^)^. Thus, age- and sex- specific criteria for obesity should be used in preschool children. Recently, detailed age- and sex-specific cutoff points for childhood obesity were proposed on the basis of an international cross-sectional survey^10^^)^. The proposed cutoff points, linked to adult cutoff points, were considered to be less arbitrary. Therefore, the use of the proposed cutoff points may be desirable for defining childhood obesity.

Furthermore, some possible lifestyle factors leading to obesity were shown not to demonstrate a dose-response relationship^4^^)^. Consequently, because 3 to 4 categories of the original lifestyle variables were dichotomized in our previous analysis, the results could lead to a masking of the real relationship between lifestyles and obesity. Moreover, lifestyles considerably change in preschool years. While typical infantile sleep consists of frequent intermittent short sleeps, amounting to more than 12 hours sleep per day, the impact of aging on sleep is characterized by a sizable long sleep and shortening of sleeping hours^11^^)^. Eating and exercising habits may change in preschool years as well. Therefore, as a possible solution for considering changes in lifestyle in preschool children, detailed age adjustments would be helpful.

In this study, we have reanalyzed the data from our initial survey using the newly proposed cutoff points for childhood obesity, the original categories of lifestyle variables, and months after birth as the variable to be adjusted in the analysis, to identify the lifestyles associated with obesity in preschool years.

## METHODS

### Subjects

The subjects included in the present study were all born in one year period between 2nd April 1989 and 1st April 1990. The survey was conducted between April 1992 and March 1994. The total study population consisted of 10,177 children. A questionnaire was distributed through regional public health centers on the day preceding the health checkup and anthropometric measurements. Of those who received a questionnaire, 9,674 parents of children (95.1%) responded to the questionnaire and anthropometric measurements were conducted. The following respondents were excluded in the analysis: those whose parental anthropometric data were not obtained (199 subjects); those who did not answer one or more questions concerning physical activity, eating habits, and sleeping habits (534 subjects). The remaining 8,941 subjects (87.9% of the total population, 4,590 males and 4,351 females) represent the study population. The mean age of the study subjects was 3.4 years and the range was 2.5 to 4.3 years.

### Questionnaire survey and anthropometric measurements

The questionnaire was filled in by parents and consisted of the following items. Eating habits included the frequency of taking breakfast and a snack and the regularity of taking snacks. Frequency of taking breakfast: rarely, almost daily, and daily. Frequency of taking a snack: 1 / a day, 2 / a day, 3 / a day, 4 or more / a day. Regularity of snacking: regular, almost regular, irregular. Exercise habits included physical activity compared to peers and time spent in outdoor playing. Physical activity compared to peers: less than, similar to, more than peers. Time spent in outdoor playing: less than 30 min, 30 to 60 min, 60-120 min, 120 min or more. Sleeping habits were categorized in the following way; wakeup time; before 6 AM, 6 to 7AM, 7 to 8 AM, and after 8 AM; bedtime: before 9 PM, 9 to 10 PM, 10 to 11 PM, and after 11 PM; sleeping hours: less than 9, 9 to 10, 10 to 11, and 11 hours or more; total hours of naps: less than 1 hour; 1 to 2, 2 to 3, 3 hours or more. The questionnaire was pretested and checked for the reproducibility of lifestyle variables at an interval of 3 months, with the use of proportion of agreement and kappa coefficients. Proportion of agreement ranged from 0.50 to 0.83. Kappa coefficients ranged from 0.48 to 0.64. Thus the questionnaire was considered to have moderate to high reproducibility^12^^)^.

Anthropometric measurements of children were undertaken at regional public health centers by trained public health nurses, according to the protocol of the Law for the Health of Mothers and Children. The heights and weights of children were measured in their shorts. Height was measured to the nearest centimetre, using a stadiometer. Weight was measured to the nearest 0.1 kg, using a weighing scale. The stadiometer was checked for accuracy and the weighing scale was calibrated before the examination. The heights and weights of parents were self-reported in the questionnaire.

We defined childhood obesity on the basis of the proposed age- and sex- specific cutoff points (17.55-18.13 kg/m^2^ for males and 17.28-17.76 kg/m^2^ for females)^10^^)^, which are linked to an adult weight of 25 kg/m^2^. Parental obesity was defined as a BMI of 25 kg/m^2^ or more based on the WHO criteria^13^^)^.

### Statistical analysis

We assessed the difference in age, BMI, and lifestyle variables between the present study subjects and those excluded from the present analysis. The unpaired t-test was used to compare the difference in age of children, child BMI, and parental BMI between the two groups. The chi-squared test was used to compare the difference in sex and lifestyle variables between the two groups.

Logistic regression analysis was performed to evaluate the strength of the relationship between parental obesity or lifestyle factors and childhood obesity. Odds ratio for childhood obesity in a category of lifestyle was calculated independently with dummy variables to the reference category.

In childhood obesity, age and sex are known to have the potential to interact with several variables^5^^)^. Therefore, we examined the interaction of each of these with all other variables in a multivariate model. The Hosmer-Lemeshow test^14^^)^ was used to validate all the logistic regression models. Statistical analyses were performed by SPSS (7.5.1J). Two-tailed *P* values of less than 0.05 were considered to be significant.

## RESULTS

We compared the difference in age, child BMI, parental BMI, and lifestyle variables between the present study subjects and those excluded from the present analysis. There were no significant differences in these variables. Thus, there appeared to be no significant selection biases in the present study.

The prevalence of obesity, the distribution of average BMI, and odds ratios (ORs) for obesity are presented in Table 1. In the multivariate analysis, age (months after birth) and sex were adjusted. Paternal and maternal obesity were strongly associated with the obesity of their children. Adjusted ORs for paternal and maternal obesity for obesity of their children were 1.74 (95% confidence interval: 1.47-2.06) and 2.62 (2.12-3.25), respectively. Rare intake of breakfast showed a non significant but lower OR for obesity. With regard to snack taking, both regularity and frequency of snack taking were not associated with obesity. Duration of outdoor playing between 60 to 120 min a day was related to significantly lower OR for obesity. The adjusted OR was 0.79 (0.64-0.99), compared to those playing for less than 30 min a day. With regard to sleeping habits, a significant increasing trend was observed for the relationship between early wakeup or short sleeping hours and obesity, while bedtime and total hours of naps were not related to obesity.

**Table 1.** The strength of parental obesity and lifestyle factors on obesity in preschool years.

	No. of subjects (n=8218) n	Obese (n=723) n	Prevalence of obesity %	Body mass index mean (SE)	Univariate OR (95%CI)	Age and sex adjusted OR (95%CI)
Paternal obesity						
non-obese	7170	513	7.2	15.87 (0.01)	1.00	1.00
obese	1771	210	14.3	16.15 (0.03)	1.75 (1.47-2.07)	1.74 (1.47-2.06)
Maternal obesity						
non-obese	8237	602	7.3	15.87 (0.01)	1.00	1.00
obese	704	121	17.2	16.48 (0.06)	2.63 (2.13-3.26)	2.62 (2.12-3.25)
Breakfast						
daily	6697	567	8.5	15.95 (0.02)	1.00	1.00
almost daily	1958	141	7.2	15.86 (0.03)	0.84 (0.69-1.02)	0.84 (0.70-1.02)
rarely	286	15	5.2	15.69 (0.08)	0.60 (0.35-1.01)	0.60 (0.35-1.02)
Regularity of snacking						
regular	748	64	8.6	15.94 (0.04)	0.98 (0.73-1.31)	0.97 (0.72-1.29)
almost regular	5526	426	7.7	15.91 (0.02)	0.87 (0.74-1.03)	0.87 (0.73-1.03)
irregular	2667	233	8.7	15.93 (0.03)	1.00	1.00
Frequency of snacking						
≦ 1 / a day	2196	174	7.9	15.94 (0.03)	1.00	1.00
2 / a day	4926	397	8.1	15.92 (0.02)	1.02 (0.85-1.23)	1.03 (0.85-1.24)
3 / a day	1629	131	8.0	15.91 (0.03)	1.02 (0.80-1.29)	1.04 (0.82-1.32)
4 / a day ≦	190	21	11.1	16.03 (0.10)	1.44 (0.89-2.33)	1.47 (0.91-2.38)
Exercise activity to peers						
more than	4922	406	8.2	15.95 (0.02)	1.00	1.00
similar to	3814	295	7.7	15.88 (0.02)	0.93 (0.80-1.09)	0.91 (0.78-1.07)
less than	205	22	10.7	16.05 (0.10)	1.34 (0.85-2.11)	1.31 (0.83-2.07)
Hours of outdoor playing						
< 30 min.	1972	166	8.4	15.96 (0.03)	1.00	1.00
30-60 min.	3579	320	8.9	15.92 (0.02)	1.07 (0.88-1.30)	1.07 (0.88-1.31)
60-120 min.	2574	174	6.8	15.89 (0.02)	0.79 (0.63-0.98)	0.79 (0.64-0.99)
120 min. ≦	816	63	7.7	15.96 (0.04)	0.91 (0.67-1.23)	0.93 (0.69-1.26)
Wakeup time						
< 6 am	93	13	14.0	16.20 (0.14)	2.24 (1.20-4.21)	2.32 (1.24-4.36)
6-7 am	2154	211	9.8	15.92 (0.03)	1.50 (1.15-1.96)	1.51 (1.15-1.98)
7-8 am	5524	420	7.6	15.90 (0.02)	1.14 (0.89-1.46)	1.14 (0.89-1.47)
8 am ≦	1170	79	6.8	15.81 (0.04)	1.00	1.00
Bedtime						
<9 pm	1269	111	8.7	16.00 (0.04)	1.00	1.00
9-10 pm	4710	389	8.3	15.92 (0.02)	0.94 (0.75-1.17)	0.95 (0.76-1.19)
10-11 pm	2598	194	7.5	15.89 (0.02)	0.84 (0.66-1.07)	0.86 (0.67-1.09)
11 pm ≦	364	29	8.0	15.86 (0.07)	0.90 (0.59-1.38)	0.91 (0.59-1.40)
Sleeping hours excluding naps						
< 9 hrs	342	36	10.5	16.10 (0.07)	1.47 (0.95-2.28)	1.52 (0.98-2.36)
9-10 hrs	3305	283	8.6	15.94 (0.02)	1.17 (0.87-1.57)	1.20 (0.89-1.62)
10-11 hrs	4539	348	7.7	15.90 (0.02)	1.04 (0.77-1.39)	1.05 (0.78-1.41)
11 hrs ≦	755	56	7.4	15.88 (0.05)	1.00	1.00
Hours of naps						
<1 hrs	5497	455	8.3	15.93 (0.02)	1.00	1.00
1-2 hrs	2784	216	7.8	15.91 (0.02)	0.93 (0.79-1.10)	0.94 (0.79-1.12)
2-3 hrs	632	47	7.4	15.89 (0.05)	0.89 (0.65-1.22)	0.91 (0.66-1.24)
3 hrs ≦	28	5	17.9	16.23 (0.22)	2.41 (0.91-6.37)	2.45 (0.92-6.49)
Sleeping hours including naps						
< 9 hrs	149	16	10.7	16.09 (0.10)	1.68 (0.97-2.92)	1.66 (0.95-2.88)
9-10 hrs	1887	170	9.0	15.96 (0.03)	1.38 (1.08-1.77)	1-38 (1.08-1.77)
10-11 hrs	5168	421	8.1	15.94 (0.02)	1.24 (1.00-1.53)	1-23 (0.99-1.52)
11 hrs ≦	1737	116	6.7	15.83 (0.03)	1.00	1.00

OR: odds ratio, 95%CI: 95% confidence interval

To clarify for the possible confounding effect of variables, we fitted a multivariate model to include the statistically significant variables presented in Table 1. The results are shown in Table2. In the construction of this model, because wakeup time was not independent of sleeping hours and sleeping hours was considered to be more important in the development of obesity, wakeup time was not included in the model. After controlling for the effects of variables, parental obesity and short sleeping hours remained significant. Compared to those taking 11 hours sleep or more, the adjusted OR was 1.20 (0.97-1.49) for those taking 10 to 11 hours sleep, 1.34 (1.05-1.72) for those taking 9 to 10 hours sleep, and 1.57 (0.90-2.75) for those taking less than 9 hours sleep. Multiplicative interaction between age, sex, and all other lifestyle variables did not add to the relationship between lifestyles and childhood obesity. The interaction of the variables paternal and maternal obesity and the interaction of the variables parental obesity and sleeping hours did not significantly add to the model. The Hosmer-Lemeshow tests supported the fit of the logistic regression model.

**Table 2.** Adjustments for parental obesity and lifestyle variables on the effects of obesity in preschool years.

	No. of subjects (n=8218) n	Obese (n=723) n	Prevalence of obesity %	Multivariate OR (95%CI)	*P* or *P* for trend
Paternal obesity					
non-obese	7170	513	7.2	1.00	
obese	1771	210	14.3	1.70 (1.43-2.02)	
Maternal obesity					<0.001
non-obese	8237	602	7.3	1.00	
obese	704	121	17.2	2.56 (2.07-3.17)	
Hours of outdoor playing					<0.001
< 30 min.	1972	166	8.4	1.00	
30-60 min.	3579	320	8.9	1.10 (0.90-1.33)	
60-120 min.	2574	174	6.8	0.80 (0.64-1.01)	
120 min. ≦	816	63	7.7	0.95 (0.70-1.29)	
Sleeping hours including naps					0.084
< 9 hrs	149	16	10.7	1.57 (0.90-2.75)	
9-10 hrs	1887	170	9.0	1.34 (1.05-1.72)	
10-11 hrs	5168	421	8.1	1.20 (0.97-1.49)	
11 hrs ≦	1737	116	6.7	1.00	0.010

OR: odds ratio, 95%CI: 95% confidence interval

In multivariate analysis, age, sex, parental obesity, hours of outdoor playing, and sleeping hours including naps were simultaneously entered. A *P* value for trend was also calculated for 4 categories of lifestyle variables modeled as a continuous variable.

We also analyzed the relationship between short sleeping hours and obesity, stratified by parental obesity, as shown in Table 3. The relationship between short sleeping hours and obesity remained significant in children with non-obese and obese parents, although no significant relationship was found in children whose father or mother was obese.

**Table 3.** The relationship between sleeping hours and obesity, stratified by parental obesity.

	Children with non-obese father and mother (n=6637)			Children with obese father and non-obese mother (n=1600)			Children with nonobese father and obese mother (n=533)			Children with obese father and mother (n=171)		
			
	Total n	Obese n (%)	OR (95%CI)	Total n	Obese n (%)	OR(95%CI)	Total n	Obese n (%)	OR(95%CI)	Total n	Obese n (%)	OR(95%CI)
sleep hours												
< 9 hrs	108	9 (8.3)	1.68 (0.81-3.48)	24	3 (12.5)	1.49 (0.42-5.32)	13	2 (15.4)	0.61 (0.12-3.08)	4	2 (50.0)	6.53 (0.69-61.7)
9-10 hrs	1390	112 (8.1)	1.61 (1.18-2.21)	341	28 ( 8.2)	0.89 (0.51-1.54)	124	15 (12.1)	0.60 (0.28-1.23)	32	15 (46.9)	4.25 (1.26-14.4)
10-11 hrs	3827	248 (6.5)	1.29 (0.98-1.71)	927	108 (11.7)	1.31 (0.85-2.04)	307	44 (14.3)	0.70 (0.37-1.32)	107	21 (19.6)	1.21 (0.40-3.65)
11 hrs ≦	1312	66 (5.0)	1.00	308	28 ( 9.1)	1.00	89	17 (19.1)	1.00	28	5 (17.9)	1.00
*P* for trend			0.002			0.791			0.199			0.004

OR: odds ratio, 95%CI: 95% confidence interval

The relationship between short sleeping hours and obesity, stratified by parental obesity. In multivariate analysis, age, sex, hours of outdoor playing, and sleeping hours including naps were simultaneously entered. A *P* value for trend was also calculated for 4 categories of the variable sleeping hours modeled as a continuous variable.

## DISCUSSION

The present study revealed that parental obesity and short sleeping hours were associated with obesity in preschool children. These results are consistent with our previous study^6^^-^^8^^)^. Furthermore, the relationship between short sleeping hours and obesity was a dose-response relationship. In contrast to our previous studies, regularity and frequency of snack taking and physical activity were not significantly associated with obesity, although subjects taking snacks more than 4 times a day and those playing outside between 60 to 120 min a day tended to have a higher prevalence of obesity. Although the reason for these discrepant results was not clear, the use of dichotomized lifestyle variables in the previous studies might be one possible explanation, because the relationship between physical activity or snack-taking and obesity was not a dose-response relationship.

In this study, parental obesity is strongly associated with childhood obesity. Previous studies have also shown that parental obesity is a strong predictor of childhood obesity^15^^,^^16^^)^. In addition to the genetic predisposition of children with obese parents for obesity^16^^)^, lifestyle factors including physical inactivity^17^^)^ and high intake of fatty foods^18^^)^ in children with obese parents could strengthen the relationship between parental obesity and obesity of their children.

The positive balance between energy consumption and expenditure is considered to be a possible reason for the increase in the number with obesity over a relatively short period of time^1^^,^^3^^-^^5^^)^. In particular, physical inactivity and long TV watching are primarily considered to contribute to positive energy balance, in recent times^3^^-^^5^^)^. In the present study, subjects playing outside less than 60 min and those with physical activity less than their peers tend to have a higher prevalence of obesity, although the above variables did not reach statistical significance. The present study is, therefore, consistent with previous studies.

In the present study, a dose-response relationship between short sleeping hours and obesity was observed. Although the biological background of the relationship between short sleeping hours and obesity is not clear, possible explanations could include the following. Firstly, growth hormone (GH) is secreted mainly in the first half of the night, in which slow wave sleep predominates in electroencephalograms^19^^)^. Shortening of sleeping hours could therefore lead to a decrease in nighttime GH secretion. Because GH plays an important role in lipolysis during the night, a decrease in GH secretion could theoretically lead to weight gain. Secondly, a recent study has revealed that short sleeping hours could lead to an increase in sympathetic nerve activity, cortisol secretion, and hyperinsulinemia, which are known to have links with the development of obesity^20^^)^. To our knowledge, two cross sectional studies have mentioned this relationship. One study, comprised of about 1000 French children aged 5 years^21^^)^, showed that subjects with short sleep of less than 11 hours was associated with obesity. The odds ratio for subjects with short sleep was 1.4 (95%CI: 1.1-1.9) after adjustment for parental obesity, TV watching, and physical activity. Another study^22^^)^, comprised of about 1800 Spanish adults, indicated that the adjusted odds ratio for obesity in subjects sleeping 9 hours or more was 0.43 (0.27-0.67), compared to those sleeping 6 hours or less. The present study also indicated that the relationship remained significant after adjustment for potential confounding factors. Furthermore, even after stratification by parental obesity, the relationship remained unchanged. Then the relationship between short sleeping hours and obesity could not be explained by the confounding effect of parental obesity and other potential lifestyle risk factors for obesity. However, because it is known that obesity is accompanied by psychiatric and somatic problems including obstructive sleep apnea syndrome (OSAS)^23^^,^^24^^)^, which could lead to sleep disturbance, we cannot deny the possibility that short sleeping hours result from obesity. However, in contrast to OSAS in adults, OSAS in children is mainly caused by adenotonsiller hypertrophy^24^^)^. Therefore, the confounding effects of OSAS on the relationship between sleeping hours and obesity may be small. Further laboratory and longitudinal studies will be required to clarify causal relationship between short sleeping hours and obesity.

In conclusion, the possible risk factors for obesity in preschool children in this study were parental obesity and short sleeping hours. In addition, the relationship between short sleeping hours and obesity was a dose-response relationship. The relationship between sleeping hours and obesity has logical limitations to the interpretation and deserves further research.
